# Human epidermal growth factor receptor 2 (HER2) and the future of bladder carcinoma

**DOI:** 10.1080/2090598X.2020.1835439

**Published:** 2020-11-09

**Authors:** Julien Sarkis, Marwan Alkassis, Joy Assaf

**Affiliations:** Department of Urology, Hotel-Dieu deFrance Hospital, Beirut, Lebanon, juliensarkis@live.com; Department of Urology, Hotel-Dieu deFrance Hospital, Beirut, Lebanon; Department of Medicine, Saint-Joseph University of Beirut, Beirut, Lebanon

Dear Editor,

In the current era of personalised cancer treatment and targeted therapy, the role of biomarkers in cancer management is becoming unquestionable. Immunotherapy is gradually revolutionising bladder cancer (BC) management with positive results from major trials in the treatment of muscle-invasive BC (MIBC) and metastatic BC [1]. Moreover, both the Southwest Oncology Group (SWOG) 8710 trial [2], as well as the more recent PURE-01 trial (ClinicalTrials.gov Identifier: NCT02736266) [3], showed a high complete response rate of nearly 40% for neoadjuvant pembrolizumab immunotherapy. However, the toxicity and the high financial cost of these drugs, plus the development of resistance to these agents, highlight the need to identify and develop prognostic and predictive biomarkers in order to maximise therapeutic effects in a small number of patients. To this extent, both United States Food and Drug Administration (FDA)-approved programmed cell death protein ligand 1 (PD-L1) and fibroblast growth factor receptors (FGFRs) have been studied as useful biomarkers for predicting the efficacy of immune checkpoint inhibitors in BC. Additionally, a recent literature review published in this journal by Nowak et al. [4] found an association between increased PD-L1 expression and BCG unresponsiveness in non-MIBC (NMIBC). Unfortunately, current evidence is inconsistent and incorporating these biomarkers into clinical practice is still premature, with further multicentre randomised trials needed to make definitive conclusions.

In this issue of the *Arab Journal of Urology*, Agrawal et al. [5] explored the prognostic and therapeutic potential of a third biomarker for BC: the human epidermal growth factor receptor 2 (HER2), a tyrosine kinase transmembrane receptor involved in cycle cell regulation and cell proliferation. While previous authors studied HER2 as a potential therapeutic target in MIBC, HER2 expression and gene amplification in NMIBC was still unclear [6]. In their retrospective analysis of 93 BCs (25 MIBC and 68 NMIBC), three results should be highlighted: (i) the HER2 protein expression by immunohistochemistry correlated with the stage and grade of BC, (ii) progression-free survival significantly correlated with stage, grade and HER2 3+ expression, and (iii) that in the NMIBC subgroup, HER2 3+ expression and gene amplification was seen only in high-grade NMIBC, all of them being lamina-invasive (pT1). Firstly, these results confirm the prognostic value of HER2, as opposed to the Kumar et al. [7] study where HER2 overexpression was not correlated with staging, lymph node metastasis or recurrence of the disease. Secondly, they showed that about one-half of non-invasive tumours show high-grade histology and HER2 3+ protein expression on immunohistochemistry, therefore paving the way for *HER2* gene amplification by fluorescent *in situ* hybridisation in all HER2 3+ high-grade NMIBC.

Clinical implications of this study are remarkable: in addition to its prognostic role in BC, HER2 amplification in MIBC and high-grade NMIBC makes HER2-targeted therapy a possible treatment modality in such patients. Multiple clinical trials using lapatinib, a potent HER1 and HER2 inhibitor, or trastuzumab, as treatments for locally advanced or metastatic BC, failed to show relevant anti-tumour activity [8,9]. But data on the efficacy of HER2-targeted therapy on high-grade MIBC is lacking and should be studied. There are actually three clinical trials evaluating the efficacy of trastuzumab (NCT04482309); PRS-343, which is a bispecific fusion protein of anti-HER2 monoclonal antibody/anti-CD137 anti-calin (NCT03330561); and PRS-343 in combination with atezolizumab (NCT03650348) in HER2-positive solid tumours, including urothelial carcinoma. These trials might give us more insight and evidence on the use of HER2-targeted therapy in treating BC.

Overall, the study here contributes valuable data in steadily improving our understanding of HER2 expression in BC. Further prospective studies, as well as ongoing trials on HER2-targeted therapy, will certainly shed more light on the role of HER2 in BC management and treatment.
